# Menstrual reform: cultural roots of delayed endometriosis diagnosis in the Asia–Pacific region

**DOI:** 10.1016/j.lanwpc.2025.101629

**Published:** 2025-06-30

**Authors:** The Lancet Regional Health – Western Pacific

The 16th World Congress on Endometriosis held in Sydney, May 21–24, put an important but often neglected women's health condition into the spotlight. Endometriosis is a chronic disease characterised by tissue similar to the endometrium growing outside the uterus, which causes debilitating menstrual pain, dysuria, and dyspareunia, alongside fertility complications. The term *pain* sometimes downplays the level of suffering among patients, as many confuse it with regular menstrual cramps despite a profound impact on quality of life and productivity—on average, endometriosis leads to 19 missed work days per year and affects one in every ten women in the world. The assumed normalcy of pain contributes to a staggering diagnostic delay of 6–8 years counting from symptom onset to definitive diagnosis, while cultural factors play a role.

In the Asia–Pacific region, deeply rooted cultural taboos have shaped how women experience menstrual ill-health and report symptoms. Menstrual blood is often viewed as dirty, cursed, and associated with misfortune and disease in southeast Asia and several Pacific island countries. In Bangladesh, all menstrual practices and discussions need to be concealed, and interactions with menstruating women are avoided. In an Indigenous Malaysian community, it is considered a polluted period of time when women are prone to evil spirits and must follow food restrictions and isolate themselves from family meals. While some practices are motivated by good intentions to protect women during a vulnerable period, they unintentionally hinder information sharing to spot symptoms. Negative beliefs could also lead to self-blaming when symptoms appear, as women may attribute abnormal pain to disobedience to culture and food rules. Even in a westernised society such as Australia, menstrual etiquette still exists to discourage open discussion of menstrual-related topics among peers, which might conceal menstrual health problems. Timely diagnosis, however, is relevant to improving patient outcomes. A study in Canada found that Asian patients who immigrated tended to be diagnosed at a more advanced stage of endometriosis than White patients in the same community, possibly due to culturally driven delay and untreated disease progression. The Asia–Pacific region urgently needs a “menstrual reform” to create an inclusive environment where women could comfortably discuss symptoms, seek professional help, and treat their needless pain earlier.

Hurdles remain as we put the vision into action. There have been public health interventions to promote awareness of menstrual disorders in communities, but the initiatives unfortunately do not always produce a lasting impact. Research indicated that information campaigns were effective in immediately improving women's knowledge, but there were gaps when translating the gain into long-term practice as the ingrained social norms and second-order beliefs—ie, the beliefs about other people's beliefs—were not easily challenged. To address this, we advocate for more culturally and locally adapted strategies to seed a new norm in these traditional societies. Apart from optimising community and school programmes, another route to start with is through primary care. Local primary care providers (PCPs) and community care workers are ideally positioned to lead this paradigm shift, as they are the first point of contact for women with severe menstrual pain. As patients consult 4.8 doctors before receiving a definitive diagnosis of endometriosis, PCPs play a crucial role in reducing diagnostic delay by collectively empowering women with accurate information and timely referral to specialist care. The cultural beliefs and attitudes of these PCPs also matter as their recognition of *chronic pain*, instead of attributing the signs to regular cramps, could directly influence patients' willingness to seek ongoing medical help. Given the information asymmetry, it is important that PCPs are trained to probe for sensitive symptoms such as sexual pain, which may be hidden by patients due to cultural appropriateness, while leading a patient-centred, respectful, and professional conversation.

Some progress towards “menstrual reform” has been seen. In 2018, Australia led the way in the Asia–Pacific region by establishing the first National Action Plan for Endometriosis to coordinate nationwide actions in awareness building, clinical management, and research. Recognising that diagnosis at the primary care level is difficult and that PCPs often face uncertainty about when to escalate pain to specialist care, Australian researchers developed a low-cost diagnostic tool named PIPPA to assist PCPs and patients with early screening, with a scoring system validated using local cohorts. Large-scale education programmes have been rolled out in secondary schools to promote correct understanding of menstrual health among adolescents of all genders, as everyone matters in shaping social norms and preventing stigma perpetuation. Additionally, Endometriosis and Pelvic Pain Clinics have been set up to streamline the referral pathway into one-stop services in primary care. In other economies, the MyEndosis campaign, introduced by a Malaysian advocacy group, has leveraged social media to connect women with menstrual pain by forming strong peer support in a safe and anonymous space without embarrassment. Other Asian–Pacific countries or regions, including Japan, South Korea, Taiwan, Indonesia, and the Philippines, also undertook substantial work to provide menstrual leave, which is a good step to recognise the presence of unbearable pain and medical need. We look forward to seeing more initiatives as well as evidence of cost-effectiveness and outcomes in shortening diagnostic delays via ongoing evaluation. The findings will facilitate cross-cultural exchange across the Asia–Pacific region to plan national actions and priorities in women's health, especially in low-resource settings and countries with diverse cultural backgrounds.

Breaking cultural barriers is always a challenging endeavour, but it is a vital step to address the diagnostic delays in endometriosis. Empowerment in primary care could be a starting point with the right tools and training to help women address painful experiences early. The progress made in individual countries offers a blueprint, but active participation from all cultures is necessary to learn from each other and drive a new norm in the Asia–Pacific region. Diagnostic delay up to 8 years is not acceptable, and we cannot afford to see the same in the next decade.

